# Recurrent de novo pathogenic variant of *WASF1* in a Japanese patient with neurodevelopmental disorder with absent language and variable seizures

**DOI:** 10.1038/s41439-021-00176-4

**Published:** 2021-11-29

**Authors:** Keiko Shimojima Yamamoto, Tomoe Yanagishita, Hisako Yamamoto, Yusaku Miyamoto, Miho Nagata, Yasuki Ishihara, Yohei Miyashita, Yoshihiro Asano, Yasushi Sakata, Toshiyuki Yamamoto

**Affiliations:** 1 Department of Transfusion Medicine and Cell Processing, Tokyo Women’s Medical University, Tokyo, 162-8666 Japan; 2 Tokyo Women’s Medical University Institute of Integrated Medical Sciences, Tokyo, 162-8666 Japan; 3 Department of Pediatrics, Tokyo Women’s Medical University, Tokyo, 162-8666 Japan; 4 Department of Pediatrics, St. Marianna University School of Medicine, Kawasaki, 216-8511 Japan; 5 Department of Cardiovascular Medicine, Osaka University Graduate School of Medicine, Suita, 565-0871 Japan; 6 Department of Legal Medicine, Osaka University Graduate School of Medicine, Suita, 565-0871 Japan; 7 Institute of Medical Genetics, Tokyo Women’s Medical University, Tokyo, 162-8666 Japan

**Keywords:** Disease genetics, Genetics research

## Abstract

A recurrent de novo pathogenic variant of *WASF1*, NM_003931:c.1516C>T [p.Arg506*], was identified in a 6-year-old female Japanese patient with severe developmental delay, hypotonia, hyperkinetic behavior, and distinctive facial features. The initial report of five adult patients with *WASF1* variants was the only previous report regarding variants of this gene; this is the second such report, reaffirming that rare but recurrent truncating variants of *WASF1* are associated with severe neurodevelopmental disorders.

In 2018, the WAS protein family member 1 (*WASF1*) gene was identified as being responsible for neurodevelopmental disorder with absent language and variable seizures (NEDALVS; MIM # 618707)^1^. This is the only existing report of the pathogenic variants of *WASF1* to date; no additional reports have been made since then. Recently, we identified the same variant of *WASF1* in a Japanese patient with severe developmental delay, seizures, and distinctive facial features.

The 6-year-old female patient had been delivered at 38 weeks of gestation, with a birth weight of 3585 g (90th–97th percentile), a length of 49.5 cm (50th–75th percentile), and an occipitofrontal circumference (OFC) of 35.2 cm (90th–97th percentile); the delivery was performed by caesarian section due to breech position. Her healthy parents were 38 years old at her birth. Her elder sister was also healthy. Thus, there was no family history of neurodevelopmental disorders. In early infancy, the patient showed frequent vomiting, which improved later. This patient’s early development was not delayed; she was able to control her head at 3 months, roll over at 5 months, and sit upright at 7 months. However, she later showed developmental delays, standing with support at 18 months and walking alone at 24 months. She showed distinctive facial features, including thin arched eyebrows, epicanthus, right internal strabismus, a flat nasal bridge, anteverted nares, a long philtrum, and a small and tented mouth (Fig. 1A). The patient has had several episodes of febrile convulsions to date; however, she has shown only one episode of convulsion without fever. Therefore, her clinical course is now being monitored without the prescription of antiepileptic drugs, although her electropherogram showed abnormalities with focal spikes.

**Fig. 1:**
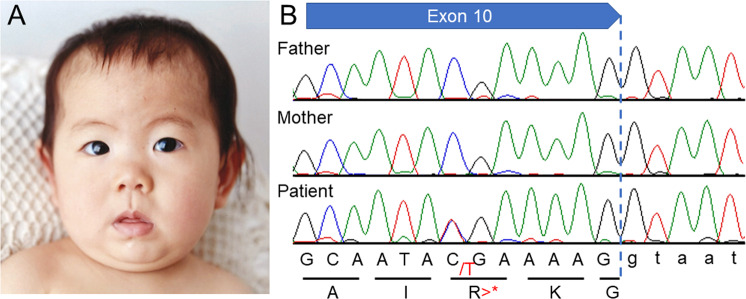
Results of this study. **A** A portrait of the reported patient taken at 1 year of age and provided by her parents with written informed consent. The portrait shows the patient’s arched eyebrows, left internal strabismus, flat nasal bridge, anteverted nares, long philtrum, and small and tented mouth. **B** Sanger sequencing shows a heterozygous variant, c.1516C>T [p.Arg506*], only in the patient and not in her parents, indicating de novo occurrence.

At present, the patient’s height, weight, and OFC are 110 cm (25–50th percentile), 19.2 kg (25–50th percentile), and 49.5 cm (10–25th percentile), respectively. She still cannot speak meaningful words, but she has gradually become able to understand the spoken language of others and is interested in letters. She attempts to convey her intentions with gestures because she cannot do so through language; if she still cannot convey her intentions, she grabs the person’s arm and takes him or her to what she wants, a behavior known as the crane phenomenon. If things do not go as she expects, she does not panic but throws a tantrum. Stereotyped behavior is not shown. She cannot remain in place during group activities. She displays hyperkinetic behavior and is constantly wandering around. She can gaze at others, but her interests tend to shift quickly; therefore, she soon redirects her gaze. She exhibits a great deal of sensory play, such as immediately putting objects she touches into her mouth. Once she puts an object into her mouth, she cannot spit it out; hence, she quickly swallows it. She cannot urinate in a toilet and always urinates in a diaper. Her mouth is always open, but she does not drool or snore during sleep. Routine blood examination revealed no abnormalities. Brain magnetic resonance imaging showed no abnormalities. Conventional chromosomal G-banding revealed a normal female karyotype of 46,XX.

Due to the patient’s unclear diagnosis, we enrolled her in the “Initiative on Rare and Undiagnosed Diseases” (IRUD) research project^2,3^. This study was performed in accordance with the Declaration of Helsinki and was approved by the ethics committee of our institution. After obtaining written informed consent, we obtained blood samples from the patient and both parents. Genomic DNA was extracted from the peripheral blood of the individuals using a standard protocol. Exome sequencing was performed using trio samples, including parental samples, as described previously. Ultimately, we identified a de novo variant, NM_003931(WASF1):c.1516C>T [p.Arg506*], which was previously reported by Ito et al.^1^. This was confirmed by standard PCR and Sanger sequencing (Fig. 1B). There were no other variants that might be related to the clinical features of this patient. The clinical features of the present patient are summarized in Table 1, combined with the clinical data provided by Ito et al.^1^. The present patient showed clinical features similar to those reported by Ito et al., although our patient is much younger than those in the previous report^1^. Therefore, we determined that the identified variant in *WASF1* is responsible for the clinical features of the present patient.

**Table 1:** Clinical features of the present patient in comparison with the previously reported patients.

	Present patient	Patients reported by Ito et al.^1^
*General*		
Age (years)	6	21–30 (average 24)
Gender	Female	4/1 (male/female)
*Birth*		
Gestation (weeks)	38	39–41
Weight (g)	3585	3370–4100 (average 3862)
Occipitofrontal circumference (cm)	35.2	35.5 (average 35.5)
*Neurological*		
Intellectual disability	Severe	Moderate to profound
Seizures	+	4/5 (onset 8 months to 8 years)
Speech	Non-verbal	1 non-verbal/3 simple words
Hypotonia	+	4/5
History of regression	−	2/5 since infancy
Wide-based gait with poor balance	−	3/5 (1/5 non-ambulant)
High pain tolerance	−	4/5 (2 of them show self-injury)
Head imaging	No abnormality	3/5 (mild atrophy or enlarged ventricules)
*Current measurements*		
Occipitofrontal circumference (cm)	49.5	2 macrocephaly/1 microcephaly
Weight (kg)	19.2	40.2–82 (average 57)
Height (cm)	110	150–183 (average 167)
*Motor development*		
Age at unsupported sitting (months)	7	6–22 (average 14)
Age at walking	24	25–120 (average 57)/1 non-ambulant
*Craniofacial*		
Midface hypoplasia	+	3/4
Strabismus	+	4/5
*Musculoskeletal*		
Joint hyperflexibility	−	3/5
Long tapered fingers	−	2/4
Ankle valgus	+	2/5
Pes planus	+	3/5
*Other*		
Widely spaced nipples	−	2/5
Café au lait macules	−	2/4
Feeding problems	+	3/5
Genitourinary	−	2/5
Constipation	−	4/5

This Table is referred to the previous report by Ito et al.^1^.

There has been only one prior report of pathogenic variants in *WASF1*, in which three different variants were identified in five different patients: c.1516C>T (p.Arg506*), c.1558C>T (p.Gln520*), and c.1482delinsGCCAGG (p.Ile494Metfs*23). *WASF1* consists of a total of 10 exons (8 coding exons), and the three previously reported de novo variants appear to cluster around the carboxy-terminal region. In particular, all variants are related to premature stop codons and are located either in the last 50-bp region of the penultimate exon or in the last exon. Thus, these transcripts will escape nonsense-mediated decay and result in the generation of a truncated protein from which the carboxy-terminal region is partially or fully absent. Ito et al.^1^ confirmed this finding using fibroblasts obtained from affected patients^1^. Therefore, it is recognized that the three reported variants possibly lead to altered function of the mutant protein rather than loss of function or haploinsufficiency due to degradation through nonsense-mediated decay, indicating that the disease has a dominant-negative mechanism.

Based on the data in gnomAD (https://gnomad.broadinstitute.org/), there are no variants related to loss of function, and the pLI is listed as 1. This may indicate that *WASF1* is intolerant to loss of function. There are some reports of interstitial deletions involving 6q21 in which *WASF1* is included^4,5^. The data in the DECIPHER database (https://www.deciphergenomics.org/) also show 6q21 microdeletions. Most patients with 6q21 deletions have intellectual disabilities. However, there are no data on chromosomal microdeletions restricted to the narrow region of *WASF1*. Therefore, we cannot precisely evaluate the phenotypic features of *WASF1* haploinsufficiency.

The initial report of five adult patients with *WASF1* variants is the only existing report concerning variants of this gene. All patients in that report were over 20 years of age, and three of them showed the same p.Arg506* variant that we observed in the present patient. They were able to sit unsupported between the ages of 9 and 18 months and began walking between the ages of 25 months and 4 years. This may indicate that their early motor development was not delayed, but the subsequent overall developmental delay became progressively more pronounced. In regard to language development, the previously reported patients could speak single words or simple sentence. Therefore, the developmental status of the previously reported patients is quite similar to that of the present patient. Four of the five reported patients showed strabismus, which was also observed in the present patient. From these findings, we concluded that the present patient had clinical features in common with the previous patients.

To date, a total of only six patients have shown pathogenic variants in *WASF1*. This indicates that pathogenic variants of *WASF1* are extremely rare. In this study, the recurrence of the de novo variant in *WASF1* was confirmed in association with severe neurodevelopmental disorders. This provides important clinical evidence of the relationship between *WASF1* and neurodevelopment.

## HGV database

The relevant data from this Data Report are hosted at the Human Genome Variation Database at 10.6084/m9.figshare.hgv.3109
